# Motivating Moral Behavior: Helping, Sharing, and Comforting in Young Children With Autism Spectrum Disorder

**DOI:** 10.3389/fpsyg.2019.00025

**Published:** 2019-01-23

**Authors:** Kristen A. Dunfield, Laura J. Best, Elizabeth A. Kelley, Valerie A. Kuhlmeier

**Affiliations:** ^1^Department of Psychology, Concordia University, Montreal, QC, Canada; ^2^Department of Psychology, Queen’s University, Kingston, ON, Canada

**Keywords:** prosocial behavior, autism spectrum disorder, moral development, social-cognitive development, helping, sharing, comforting

## Abstract

This exploratory study examined the role of social-cognitive development in the production of moral behavior. Specifically, we explored the propensity of children with Autism Spectrum Disorders (ASD) to engage in helping, sharing, and comforting acts, addressing two specific questions: (1) Compared to their typically developing (TD) peers, how do young children with ASD perform on three prosocial tasks that require the recognition of different kinds of need (instrumental, material, and emotional), and (2) are children with ASD adept at distinguishing situations in which an adult needs assistance from perceptually similar situations in which the need is absent? Children with ASD demonstrated low levels of helping and sharing but provided comfort at levels consistent with their TD peers. Children with ASD also tended to differentiate situations where a need was present from situations in which it was absent. Together, these results provided an initial demonstration that young children with ASD have the ability to take another’s perspective and represent their internal need states. However, when the cost of engaging in prosocial behavior is high (e.g., helping and sharing), children with ASD may be less inclined to engage in the behavior, suggesting that both the capacity to recognize another’s need *and* the motivation to act on behalf of another appear to play important roles in the production of prosocial behavior. Further, differential responding on the helping, sharing, and comforting tasks lend support to current proposals that the domain of moral behavior is comprised of a variety of distinct subtypes of prosocial behavior.

## Introduction

Humans are a hyper-social species. Other-regarding concerns permeate most human interactions and social structures. Indeed, the ability and willingness to act on behalf of others has important implications for well-being in contexts that range from children’s successful entry into peer culture (Wentzel, 2014), to the functioning of society as a whole (Tomasello, 2009). Over the last decade, there has been considerable interest in identifying the developmental origins of our tendency to act in ways that benefit others, potentially at a cost to ourselves.

The term *prosocial behavior* typically refers to *any* action one individual engages in to benefit another (Hay, 1994). Though this definition hints at the diversity of actions that fit this characterization, it largely treats prosocial behavior as a unitary construct requiring a single developmental explanation. Importantly, this broad definition has led to mixed success determining when prosocial behaviors first emerge (Dunfield et al., 2011), the developmental trajectories that prosocial behaviors follow (Dunfield and Kuhlmeier, 2013), what neural and behavioral correlates support its production (Paulus et al., 2013; Steinbeis, 2018), and how individual differences affect its production (Beier et al., 2018; Schachner et al., 2018; see Eisenberg et al., 2015b for a recent, broad review). As such, there has been a move to clarify the varieties of ways people can act on behalf of others and identify the unique constraints imposed by each type of prosocial response. Importantly, as the field moves toward a more nuanced understanding of the factors that support the early development of prosocial behavior, there is still striking homogeneity in the participants studied (largely neurotypical participants from WEIRD cultures: e.g., Eisenberg et al., 2015b). The current research explores early prosocial behavior in a unique participant population, namely individuals diagnosed with Autism Spectrum Disorder (ASD).

Autism Spectrum Disorder is a neurobiological disorder characterized by impaired social behavior, communication, and language difficulties, in addition to restricted, repetitive behaviors and/or interests (American Psychiatric Association, 2013). Children with ASD show reduced attention to, and gain less reinforcement from, shared social attention and interactions (Dawson et al., 2004), which is thought to result in impairment in social cognition more broadly (Chevallier et al., 2012). Specifically, the ability to recognize and understand others’ mental (theory of mind) and emotional (affect recognition) states appears delayed in children with ASD (e.g., Charman et al., 1998; Dyck et al., 2001). Thus, exploring the prosocial tendencies of young children with ASD presents a unique opportunity given documented deficits in social cognition and questions regarding the social motivation of affected individuals.

### Varieties of Prosocial Behavior

Much prosocial behavior, especially early in development, involves intervening when another individual is experiencing a problem. Effectively intervening on behalf of another requires the ability to take their perspective and notice that they are having trouble, the recognition of the cause of the problem, and the motivation to act to resolve the problem. If one fails to navigate any of these three challenges, an effective prosocial behavior is unlikely to be produced. With these constraints in mind, there are at least three varieties of negative states that individuals are likely to face and regularly resolve for others, namely, instrumental need, material desire, and emotional distress.

#### Helping

*Instrumental need* occurs when an individual is unable to complete goal directed behavior. Helping is the term we use to refer to other-oriented acts aimed at alleviating another’s instrumental need. In the now classic out-of-reach helping paradigm (Warneken and Tomasello, 2006), toddlers observe an experimenter hanging clothes on a line. As the experimenter works through their chore, they drop a clothespin where they cannot reach it, giving the child an opportunity to help by retrieving the required item.

#### Sharing

*Material desire* occurs when an experimenter does not have access to a desired resource. Sharing refers to behaviors intended to alleviate material desire in another by relinquishing control of a good. Children’s responses to material desire have been assessed in a variety of ways that range from naturalistic observations (e.g., Hay et al., 1999) to structured economic-style games (e.g., Fehr et al., 2008). Typically, children are placed in situations of either resource abundance or scarcity and often explicitly prompted to make a decision about how available resources should be distributed.

#### Comforting

Finally, *emotional distress* occurs when an individual is experiencing negative arousal and can be resolved through comforting. Comforting has been defined and examined in a number of ways that range from assessing children’s concerned attention toward emotional displays (e.g., Spinrad and Stifter, 2006) to children’s ability to approach and offer physical comfort to a distressed individual (e.g., Svetlova et al., 2010). In the current study, to facilitate comparisons across tasks, we will focus on overt responses to other’s negative emotions such as verbal (e.g., kind words) or physical (e.g., pats, hugs, or kisses) behaviors instead of more ambiguous responses such as concerned attention, which could reflect either personal distress or other-oriented concern (Eisenberg et al., 1991).

### Prosocial Behavior in Typically Developing Children

Importantly, because responding to each of these distinct needs requires different initial assessments, and the underlying social cognitive abilities emerge at different ages, we should not necessarily predict consistency regarding when in development each of these behaviors will occur nor how individual differences will affect each variety of behavior. Previous research on children’s social cognitive development suggests that within the first year of life infants can interpret goal directed action (e.g., Woodward, 1998), differentiate between intentional and accidental outcomes (e.g., Behne et al., 2005), and shortly thereafter begin to correct unintended outcomes (e.g., Meltzoff, 1995); suggesting that around their first birthday infants have the representational capacity to recognize and respond to instrumental needs. Consistent with this proposal, helping has been observed as early as 14-months (Warneken and Tomasello, 2007) and is enacted robustly in a variety of circumstances by 18-months (Warneken and Tomasello, 2006).

Sharing, on the other hand, involves the recognition of and response to material desire. Previous research reveals that, infants prefer equal distributions (and distributors; e.g., Geraci and Surian, 2011; Schmidt and Sommerville, 2011) and begin to offer others goods within the first year of life (Hay et al., 1999). However, true sharing, in which the good is given up entirely, does not emerge consistently until closer to the third birthday and then, typically only when others make their desire explicit (Brownell et al., 2013), the cost of sharing is low (e.g., Thompson et al., 1997; Moore, 2009), or the recipient is familiar (e.g., Hay, 1979; Paulus, 2016). Thus, the real challenge in addressing material desire may be the motivation to relinquish control of a desired good (e.g., Svetlova et al., 2010).

Finally, *comforting* involves the recognition of and response to a negative affective state. A major challenge associated with the production of comforting behavior is taking another’s perspective and *determining an appropriate response* in an emotional domain. Relative to the other varieties of prosocial behavior, comforting has a longer history of theoretical consideration. In what is still a dominant perspective on the development of comforting, Hoffman (1982) proposed that comforting develops over four stages with increasing complexity ranging from simple emotional contagion in infancy to veridical empathic responses that emerge closer to the fourth birthday. Consistent with this proposal, the earliest instances of “comforting” typically involve measures of concerned attention as opposed to other-oriented actions (Spinrad and Stifter, 2006), and the ability to perceive emotional distress and respond to it is affected by the types of comfort one has experienced over the course of their early life (e.g., Johnson et al., 2013; Dunfield and Johnson, 2015; Gross et al., 2017; Beier et al., 2018).

By distinguishing between the three varieties of negative states and focusing on the initial assessment the child is forced to make, researchers have demonstrated unique ages of onset, with helping and sharing preceding comforting (Svetlova et al., 2010; Dunfield et al., 2011; Paulus et al., 2013), unique developmental trajectories and uncorrelated patterns of production (Dunfield and Kuhlmeier, 2013; Sommerville et al., 2013; Eisenberg et al., 2015a), and variability associated with individual differences (Beier et al., 2018; Knafo-Noam et al., 2018; Schachner et al., 2018). These findings are consistent with the idea that varieties of other oriented behavior show distinct developmental trajectories due to the differential development of the underlying social cognitive abilities (e.g., Dunfield, 2014; Paulus, 2018). Importantly, because the production of prosocial behavior is thought to require the coordination of social understanding and motivation, critical insights can be gleaned from exploring these behaviors in atypical developmental samples.

### Prosocial Behavior in Children With ASD

Previous research reveals that some of the social cognitive abilities that underlie successful prosocial behavior are intact in young children with autism. For example, children with ASD appear to understand others’ actions on objects (Aldridge et al., 2000; Carpenter et al., 2001), suggesting that they may be able to represent other’s instrumental needs. In contrast, documented deficits in effortful control – particularly when inhibiting a prepotent response (Hill, 2004) – may make it difficult for children with ASD to relinquish control of a resource in order to alleviate another’s material desire. Further, previous research has demonstrated that impairments in emotion recognition throughout the lifespan may make the production of an effective comforting response uniquely difficult (see Bons et al., 2013 for a review). Taken together, there is reason to believe that social cognitive deficits associated with ASD may differentially impact an individual’s ability and willingness to act on behalf of another.

Few studies have examined the prosocial abilities of children with ASD. When assessed using the Strengths and Difficulties Questionnaire, parents and teachers reported reduced prosocial behavior in children and adolescents with ASD relative to typically developing (TD) participants (Iizuka et al., 2010; Jones and Frederickson, 2010; Russell et al., 2012). When assessed experimentally, 10–13 year-old children with ASD were found to engage in simple helping and sharing but at a lower rate than developmentally delayed control groups (Sigman et al., 1999; Travis et al., 2001). Importantly, these studies examined a either a single prosocial behavior, or combed them into a single prosocial score, making it impossible to separately consider the two varieties of prosocial behaviors, which may develop independently and, thus, may have occurred with different frequency. Moreover, these studies examined school-aged children, and compared to a Developmentally Delayed (DD) control group, their findings cannot speak to the emergence of prosocial behavior or speak to differences related to TD participants.

In relation to comforting, Sigman et al. (1992) reported 3.5 year-old children with ASD’s behavioral responses to another’s distress, with the highest rating being “intense affective involvement and/or comforting behavior.” Only 10 and 6% of children with ASD were rated as comforting a parent and experimenter, respectively (Sigman et al., 1992). Further, when assessing 6- and 7-year-old children with ASD’s responses to another’s emotional distress using a combination of questionnaires and online prosocial tasks, Deschamps et al. (2014) found that although children with ASD struggled with cognitive empathy (i.e., attributing a mental state to another) and social responsiveness relative to TD children, they showed similar performance on measures of affective empathy (i.e., experiencing an emotion congruent with another’s experience) and computer-mediated prosocial behavior. Importantly, deficits in concern for others appear to develop early. Indeed, by 20-months- of-age, children at risk for developing ASD are already showing diminished concern for others relative to TD peers (Charman et al., 1997).

More recently, two experimental studies have examined the ability of young children with ASD to engage in a variety of prosocial behaviors. Paulus and Rosal-Grifoll (2017) compared helping and sharing in 3- to 6-year-old TD participants and participants with ASD. To assess helping, participants watched as an experimenter accidentally knocked a jar of pens onto the floor as they left the testing room. Sharing was assessed using a resource allocation paradigm in which participants were given 10 stickers to distribute between themselves and two hypothetical recipients (one rich and one poor). The authors report higher rates of helping in the ASD population than in the TD population. Further, while TD participants shared resources equally, ASD participants tended to give the majority of their resources away. Importantly, though this study demonstrates surprising prosocial abilities in young children with ASD, it is unclear to what extent participants were motivated by the recipient’s need. Specifically, neither task included a control condition, leaving open the possibility that the participants were enacting a learned script (e.g., if things are on the floor, pick them up; see Warneken and Tomasello, 2006). Relatedly, because participants had to infer the correct response either in absence of the recipient (i.e., the helping task), or in absence of any non-verbal cues to need (i.e., sharing with cardboard cut outs), it is difficult to determine the extent to which participants are responding to the other’s needs.

Most relevant to the current study, Liebal et al. (2008) presented both 2- to 5-year-old children with ASD and a DD control group the opportunity to help and to cooperate with an experimenter. The helping task replicated the design of Warneken and Tomasello (2006) and included an experimental (need present) and control (need absent) condition. Though children with ASD clearly recognized and responded to another’s need (i.e., retrieving the object more frequently in the experimental as opposed to control condition), participants with ASD were less likely to help than their DD counterparts. Together, the extant literature suggests that children with ASD have the ability to recognize and respond to each of the three types of negative states. However, it is not clear when these abilities emerge or how frequently these behaviors are produced relative to each other, and importantly, relative to TD children.

### The Current Study

The current, exploratory study will contribute to our understanding of prosocial abilities in children with ASD by addressing two fundamental questions: (1) across the three varieties of prosocial behavior, are children with ASD adept at distinguishing situations in which an adult needs assistance from perceptually similar situations in which the need is absent?, and (2) compared to TD peers, how do children with ASD perform on tasks that require the recognition of the three different types of need (i.e., instrumental need, material desire, and emotional distress)? We recruited ASD participants with a non-verbal mental age of 3 years to facilitate meaningful comparison with Liebal et al. (2008) and because previous research suggests helping, sharing, and comforting are all within the behavioral repertoire of 3-year-old TD children when presented with similar tasks (Dunfield et al., 2011; Dunfield and Kuhlmeier, 2013). Because of the exploratory nature of this research, and the fact that children with ASD have *both* social cognitive and motivational deficits that may impede the production of prosocial behavior, the mechanism underlying any group differences in the production of prosocial behavior will be difficult to interpret. We hypothesize that, due to impaired social motivation, children with ASD will produce less prosocial behavior than their TD peers across all tasks. Moreover, because the different varieties of prosocial behavior impose different cognitive constraints, children with autism may show patterns of production that vary across tasks and differ from the developmental trajectories we have previously observed in TD children (e.g., with helping emerging first followed by sharing then comforting). Should participants with ASD show a unique pattern of production of helping, sharing, and comforting, it could highlight important avenues for new research into the ways in which social cognitive development affects the production of prosocial behaviors.

## Materials and Methods

### Participants

Twenty-eight children participated in this study. Our sample consisted of 14 children with a diagnosis of ASD and 14 typically developing children (TD; see Table 1 for details regarding the two samples). Participants in the ASD group had been formally diagnosed with ASD by a pediatrician and/or a psychologist based on the DSM-IV criteria. Diagnoses were confirmed in our lab using the Autism Diagnostic Observation Schedule (ADOS)^1^. All ASD participants met the criteria for ASD with eight participants meeting the more stringent cutoff for Autism. Participants in the TD group had no history of medical or developmental diagnoses, nor did they have a family history of ASD. Groups were matched on non-verbal mental age because the prosocial assessment did not necessarily require verbal output and previous research suggests verbal mental age may underestimate the abilities of children with ASD (Burack et al., 2002). Six additional children with ASD were excluded from the final sample due to their chronological and/or mental ages exceeding the testing age range (*n* = 4) or failure to confirm ASD diagnosis using the ADOS (*n* = 2). No TD participants were excluded. Participants were recruited from a small southeastern Ontario city and spoke English as their primary language.

**Table 1:** Participant sample information.

	ASD		TD	
	
	Mean (*SD*)	Range	Mean (*SD*)	Range
Chronological Age	46.35 (11.33)	28–68	34.64 (17.40)	17–69
Non-verbal Mental Age^a^	39.43 (12.86)	20–60	39.12 (13.63)	19–60
Expressive Language^a^	32.85 (13.75)	12–60	35.85 (16.27)	14–60
Receptive Language	35.38 (13.05)	14–55	39.29 (13.91)	19–69
Gender	14 males		11 males, 3 female	
ADOS score^b^	13.71 (3.45)	8–18		


*^a^Groups showed no statistical differences. ^*b*^ADOS score: Autism cut-off minimum score of 12 (Module 1 and 2), Autism Spectrum Disorder cut-off minimum score of 8 (Module 2), or 7 (Module 1). ^*1*^All children had an existing diagnosis of ASD upon participation in our study. Diagnoses were then confirmed using the Autism Diagnostic Observation Schedule (ADOS). Given that diagnostic stability of an ASD is questionable during the preschool years (Kleinman et al., 2008) and that the ADOS is not effective at differentiating between diagnoses on the spectrum (e.g.,McConachie et al., 2005), we elected to include children with a diagnosis anywhere on the autism spectrum. According to ADOS scores, 8 children with ASD exceeded the cutoff for Autism, and the remaining 6 children met only the ASD cutoff score.*

### Measures

#### Autism Diagnostic Observation Schedule (ADOS; Lord et al., 2002)

The ADOS is a standardized tool used to evaluate and diagnose children on the autism spectrum. All participants with ASD participated either in Module 1 or 2 of this test to confirm their existing diagnoses.

#### Mullen Scales of Early Learning (MSEL; Mullen, 2005)

The MSEL is a standardized test to evaluate the development (language, motor, visual abilities) of children from birth through age 69 months. Given that the tasks included in the present study were largely non-verbal, age-equivalent scores on the visual reception domain (an indicator of non-verbal IQ) were used to individually match participants from each group on mental age. That said, all children also completed the language subscales of the MSEL and there were no significant differences in language abilities across groups.

### Procedure and Design

Participation involved two visits for children with ASD and, in general, one visit for TD children, with each session lasting approximately 45 min. Four TD children required two visits due to their involvement in an additional study; these children engaged in the experimental task during the first visit and the MSEL during the second. The remaining TD children completed the experimental procedure, followed by the MSEL in a single visit. Nine of the children with ASD also participated in an additional study. Most children with ASD (*n* = 9) completed the experimental procedure and the MSEL in one visit and the ADOS in the other. On average, the timeline between children’s first and second visits was 3 weeks (*M* = 21.88 days, *SD* = 26.92).

In order to explore the prosocial tendencies of children with ASD, participants engaged in a play-based experiment that assessed their responses to instrumental needs, material desires, and emotional distress. In addition, joint attention, intention understanding, and imitation were assessed but are not reported here. Interspersing prosocial trials within other tasks allowed us present the prosocial tasks in a manner that appeared credible and somewhat natural. Caregivers who opted to accompany their child into the testing room were seated behind the participant and instructed not to influence or encourage their child’s responses toward the experimenter; however, they were allowed to comfort their child if the child approached them in distress.

#### Prosocial Assessment

Replicating Dunfield et al. (2011), children were presented with two opportunities to engage in each of the three varieties of prosocial behavior (helping, sharing, and comforting). For each of the three negative states, children were presented with two varieties of trials. In the experimental condition, the experimenter demonstrated her negative state (e.g., outstretched arm) whereas in the control condition the experimenter engaged in a perceptually matched display that did not demonstrate need (e.g., placing the toy on the ground). By attempting to match the two displays as closely as possible we can ensure that any differences between the two conditions reflect specific responses to the observation of need. The control trial for a specific prosocial task never immediately followed or preceded the corresponding experimental trial. The order of presentation of experimental and control prosocial trials was counterbalanced in four orders such that for half of the participants, the experimental trial was seen before its respective control trial. In all experimental prosocial trials, the experimenter never verbally requested aid.

#### Instrumental Need

Helping was elicited using an “out of reach” task that conceptually replicated the “clothespin” task from Warneken and Tomasello (2006). In this task, an experimenter (E1) picked up a small plastic toy and playfully walked it across the table. In the experimental condition, she dropped they toy over the edge of the table and said “oops!” while reaching for it. E1 reached with an outstretched arm and hand gesturing toward the toy for 5 s, and she then alternated her gaze between the toy and the child for 5 s until the participant provided a response or the trial ended (i.e., 10 total seconds had elapsed). In the control condition, E1 deliberately placed the toy on the floor and said “there!”, folding her arms on the edge of the table. E1 held this pose with a neutral expression for 10 s or until the child provided a response. Trials ended when 10 s elapsed or the children engaged in helping behavior, which consisted of retrieving the toy and giving it to E1. Observed non-target behaviors included ignoring the toy, playing with the toy, or explicitly refusing to assist the experimenter.

#### Material Desire

Sharing was elicited using an “unequal snack” task (Dunfield et al., 2011). Prior to sharing trials participants were told that they would be getting a snack. A second experimenter (E2) entered the testing room with two small plastic containers that contained either cheese flavored or graham crackers (based on the parent’s prior selection). E2 always offered E1 her snack first, holding the container out so both the participant and E1 could see the contents. When E1 received her snack she showed the contents to the participant and remarked, “Look what I have.” After observing E1’s snack, the child was given their container. In experimental trials, E1 received no treats while the participant received four. E1 made a sad face and placed a hand outstretched, palm up in a requesting gesture. She gazed down at her hand for 5 s then alternated her gaze between her hand and the participant for 5 s. In the control condition, both E1 and the participant received two treats. E1 waited for the child to receive their treats before she began consuming hers while gazing at the child with a neutral expression. Trials ended when the participant shared by offering E1 one or more of their treats (i.e., they engaged in prosocial behavior), failed to share by consuming all their treats, explicitly denying the experimenter (i.e., saying no or picking up the treats and creating distance), or 10 s elapsed.

#### Emotional Distress

Comforting was elicited using a “physical harm” task (Dunfield et al., 2011). In this task E1 banged her knee against the edge of a low table making a loud noise. In the experimental condition, E1 then sat down with a look of distress on her face and rubbed her knee while vocalizing pain “oh! my knee, I banged my knee!”. For the first 5 s the experimenter looked down at her knee, and then for the next 25 s she alternated her gaze between her knee and the participant. In the control condition, the experimenter simply sat down and looked toward the participant for 30 s with a neutral expression on her face. Because previous research suggests that 10 s may not provide enough time for the participants to respond to emotional distress (see Discussion, Dunfield et al., 2011), we report two comforting assessments. First, to allow comparisons to other tasks, we report participants’ responses following 10 s of emotional distress. Second, we report participants’ responses to emotional distress over a 30 s period. Trials ended when the participant comforted the experimenter or 30 s elapsed.

To account for the diversity of appropriate comforting responses, in both the experimental and control condition, children were evaluated for their engagement in approach comforting behavior (i.e., walks over to the experimenter to see if she is alright, kisses experimenter’s knee) and non-approach comforting behavior (i.e., vocalizing concern for the experimenter from a distance, providing instructions for help, directing the caregiver’s attention toward the experimenter to help her). Importantly, for instances of both approach and non-approach behavior, only behavior aimed at alleviating the distress of the experimenter were considered ‘comforting.’ Vocalizations and approaches that did not serve to provide comfort were coded as non-target behavior. Other non-target behaviors included approaching the caregiver without drawing attention to E1 (e.g., self-soothing), ignoring or failing to respond to E1 at all, and actively refusing to assist her.

For each of the prosocial tasks, regardless of condition, participants received a score of 1 if they produced the target behavior and a score of 0 otherwise. Two ASD participants did not receive all six prosocial trials due to their disengagement from testing: one participant did not provide data for a comforting control trial and both sharing trials, and the other participant did not provide data for a helping control trial and two sharing trials. As a result, 13 ASD participants were included in the helping and comforting analyses and 12 ASD participants were included in the sharing analysis.

### Coding and Data Analysis

Each session was coded by a research assistant who was blind to the purpose and hypotheses of the study. A second blind coder coded a subset of the videos (10 videos, 35%) to measure inter-rater reliability. Inter-rater agreement ranged from strong to almost perfect across all prosocial tasks (Helping: Experimental *κ* = 1.00, Control *κ* = 1.00; Comforting: Experimental *κ* = 0.86, Control *κ* = 1.00; Sharing: Experimental *κ* = 1.00, Control *κ* = 1.00; McHugh, 2012).

## Results

Due to the predominance of male participants, we did not assess gender as an independent variable. Additionally, because of the small sample and four counterbalanced orders there was not sufficient power to determine if order effected participants’ responses. Importantly, previous research employing the same tasks and design did not observe effects of gender or order, suggesting these variables are unlikely to have a meaningful effect in the current sample (see Dunfield et al., 2011).

### Instrumental Need

**FIGURE 1:**
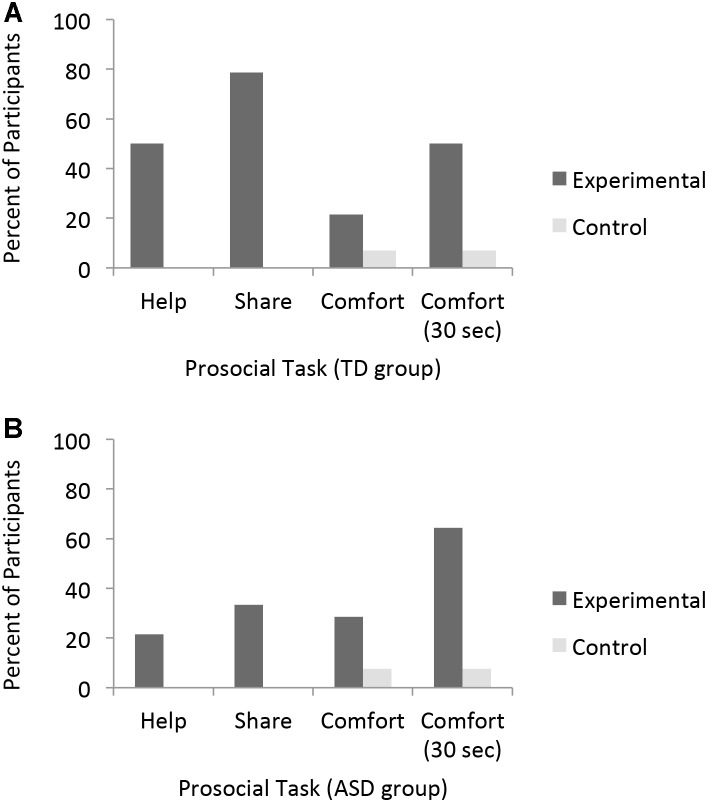
Percent of Typically Developing participants **(A)** and participants with ASD **(B)** who responded to instrumental need, material desire, and emotional distress by condition. All Participants were given up to 10 s to respond except where noted (i.e., Comfort 30 s).

**FIGURE 2:**
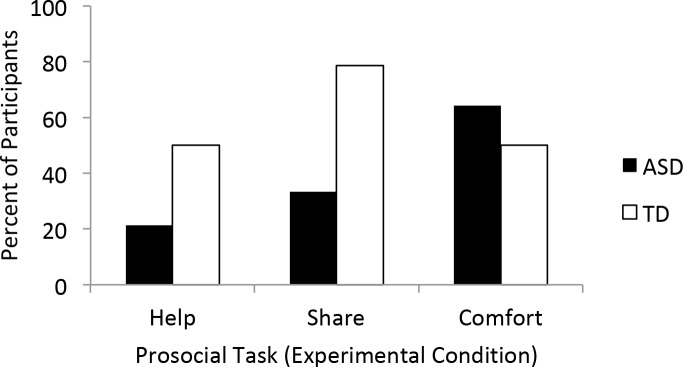
Comparison of prosocial behavior in the experimental condition across the three varieties of need (instrumental, material, and emotional) by group. These bars reflect 10 s response periods for helping and sharing and a 30 s response period for comforting.

To assess children’s responsiveness to an experimenter’s instrumental need, we compared the frequency of helping across the experimental and control condition. If participants are sensitive to the experimenter’s need they should help more frequently in the Experimental than Control condition. TD participants helped more in the experimental versus control condition (McNemar test, 1, *N* = 14, *p* = 0.02, Figure 1A) whereas, participants with ASD were equally unlikely to return the toy in both conditions (McNemar test, 1, *N* = 13, *p* = 0.25, Figure 1B). In the experimental condition, seven TD participants (50%) helped, in contrast to three participants with ASD (21.4%). Importantly, no participants from either group retrieved the toy in the control condition. Although the ASD participants did not help significantly more in the experimental condition than the control condition, the number of ASD participants offering help in the experimental condition was not significantly different from the number of TD participants who helped in the experimental condition (χ^2^= 2.49, 1, *N* = 28, *p* = 0.12; Figure 2).

### Material Desire

Comparing the frequency of sharing across the Experimental and Control condition assessed children’s response to material desire. Children who are sensitive to another’s material desire are expected to share more in the Experimental than Control condition. TD participants shared more in the experimental condition than control condition (McNemar test, 1, *N* = 14, *p* = 0.001, Figure 1B). Eleven TD participants (78.6%) offered E1 treats in the experimental condition whereas no participants offered their treats in the control condition. In contrast, ASD participants did not share more frequently in the experimental condition (4, 33.3%) than in the control condition (0, 0%; McNemar test, 1, *N* = 12, *p* = 0.12, Figure 1B). When comparing sharing rates in the experimental condition across the two groups, TD participants were significantly more likely to share than participants with ASD (χ^2^= 5.42, 1, *N* = 26, *p* = 0.02; Figure 2).

### Emotional Distress

Children’s sensitivity to emotional distress was assessed by comparing comforting behavior in the presence (Experimental) or absence (Control) of distress cues. Children who are sensitively responding to another’s distress are expected to comfort more in the experimental than control condition. Importantly, previous research suggests that children may take longer to respond to emotional cues relative to instrumental need or material desire. To that end, we are reporting two comforting analyses. First, in order to facilitate comparison with the other prosocial tasks, we will report comforting following a 10 s response window. Second, to ensure responses aren’t underestimated due to a short response window, we will report comforting behavior following 30 s.

Within the first 10 s, participants in both groups offered little comfort and did not differentially comfort across the two conditions (TD: McNemar test, 1, *N* = 14, *p* = 0.63; ASD: McNemar test, 1, *N* = 13, *p* = 0.50). However, when assessed over the full 30-s, both groups comforted more in the experimental than control condition (TD: McNemar test, 1, *N* = 14, *p* = 0.03, Figure 1A; ASD: McNemar test, 1, *N* = 13, *p* = 0.02, Figure 1B). Half of the participants in both groups offered comfort in the experimental condition (TD: 7, 50%; ASD: 9, 64.3%; Figure 1), whereas only one ASD and one TD participant offered comfort in the control condition (ASD: 7.7%; TD: 7.1%). The two participants who comforted in the control condition also comforted in the experimental condition. The ASD participant comforted within the first 10 s, the TD participant did so after 10 s but before the response period ended. Both groups of participants offered comfort at equally high rates following the 30 s response period (χ^2^= 0.58, 1, *N* = 28, *p* = 0.45; Figure 2).

### Relations Between Prosocial Tasks

**FIGURE 3:**
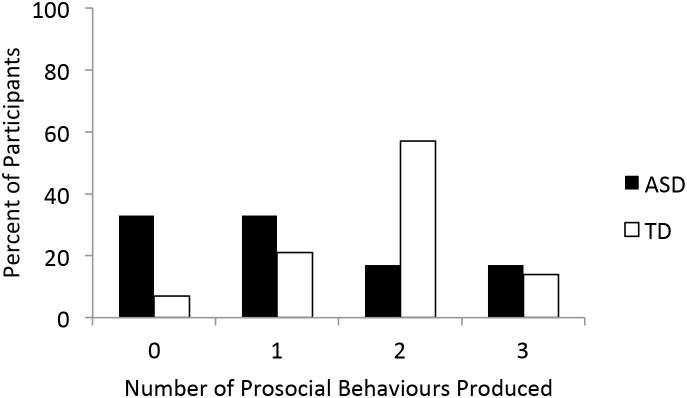
Number of prosocial behaviors produced (out of three) by group.

The majority of TD participants produced two prosocial behaviors (8, 57.1%) whereas the majority of participants with ASD produced none (4, 33.3%) or one (4, 33.3%), though the distribution of number of prosocial behaviors produced did not differ across the two groups (χ^2^= 5.42, 3, *N* = 26, *p* = 0.14; Figure 3). In the TD group, none of the prosocial tasks were associated (Φ’s -0.17 to 0.17, *p*’s > 0.51). In contrast, in the ASD group helping and sharing were associated with each other (Φ = 0.82, *p* = 0.005) but not with comforting (Φ’s < 0.24, *p*’s > 0.48). Importantly, the relation between helping and sharing in the ASD group is more likely due to the infrequency with which either behavior was produced than an actual relation between the tasks.

## Discussion

The goal of the present study was to explore the prosocial tendencies of young children with ASD by presenting tasks that involved recognizing and responding to three different varieties of need: instrumental, material, and emotional. Specifically, we examined the ability of children with ASD to distinguish situations in which an adult needs assistance (experimental condition) from perceptually similar situations in which needs are absent (control condition). We further explored prosocial motivation by comparing the frequency with which children with ASD responded to instrumental need, material desire, and emotional distress relative to mental-age-matched TD peers. We found that despite well-documented social cognitive impairments, young children with ASD were often willing and able to engage in appropriate prosocial behavior. Like their TD peers, children with ASD differentiated situations in which a need was present from situations in when a need was absent insomuch as prosocial behavior was observed only once over the course of 39 control trials.

Importantly, the picture of competence is somewhat more complicated. Though children with ASD never offered assistance when it was *not* required, they also rarely offered assistance when it *was* required. There was a trend toward offering significantly more assistance in the experimental over control conditions in response to both instrumental need and material desire; however, the difference only reached statistical significance for emotional distress. Further, when comparing the frequency with which children with ASD and TD children produce prosocial behavior, we found similar rates of helping and comforting but reduced rates of sharing. Finally, when we examined the varieties of other-oriented behaviors produced, the majority of participants with ASD produced none or one prosocial behavior, whereas the majority of TD participants produced two. Interestingly, though none of the varieties of prosocial behaviors were associated in the TD participants, the low frequency of helping and sharing lead to an apparent correlation in children with ASD. What can we make of this unique pattern of production of helping, sharing, and comforting in young children with ASD and how does it help us understand the role of social cognition and motivation in the production of early prosocial behaviors?

Below, we will review the findings for each of the three varieties of prosocial behavior in relation to past research and then interpret our current results in light of theoretical proposals regarding the nature of early prosociality. Throughout, given the exploratory nature of this research, we will highlight future research directions suggested by these results. In general, these findings support and expand existing research by demonstrating that despite marked social impairments, children with ASD *do* act prosocially in response to other’s needs and can do so in situations that extend beyond helping and cooperating (Liebal et al., 2008; Paulus and Rosal-Grifoll, 2017). Moreover, when we compare the pattern of responses across the TD and ASD participants, our results support the proposal that different types of prosocial acts are best understood as unique behaviors that depend on distinct social cognitive skills and motivations rather than a homogenous family of actions, as the amount of the prosocial behavior displayed, and relations between tasks, varied depending upon the kind of need that the experimenter was displaying and the participant’s group status.

### Helping

Consistent with past research, TD participants readily recognized and responded to an experimenter’s instrumental needs, yet they did so at a surprisingly low frequency relative to typical helping rates in methodologically similar studies (Dunfield et al., 2011; Dunfield and Kuhlmeier, 2013). Specifically, though Dunfield et al. (2011) found a similar rate of helping (50%) in 24-month olds assessed using the identical experimental paradigm, the vast majority of 2- to 4-year-old offered help in a highly similar task that afforded multiple helping opportunities (Dunfield and Kuhlmeier, 2013). Importantly, children with ASD engaged in similarly low levels of instrumental helping, with less than a quarter of affected children retrieving the out-of-reach toy for the experimenter. When looking at the overall rate of helping, the frequency with which our ASD group responded to instrumental need is approximately half that seen in the most comparable published study (Liebal et al., 2008). However, the number of children who helped exclusively in experimental trials was comparable across studies (about 20% of children with ASD in both cases). Two key methodological differences may account for the lower levels of helping observed in our sample. First, we included only one experimental trial, affording participants fewer opportunities to demonstrate their helpful capabilities. The different frequency of help offered across the two variants of Dunfield and colleagues’ past work suggests this as a viable interpretation. Further, Liebal et al. (2008) reported the portion of *trials* (out of two) that participants responded to whereas we report proportion of *participants* responding. The divergent pattern of results suggests an important avenue for examining individual differences in the tendency to produce even the simplest prosocial behaviors, especially in atypical populations (Schachner et al., 2018).

Second, the behavior displayed by the ASD group suggests that our choice of stimuli may have reduced their likelihood of helping. Specifically, the provision of instrumental help appeared to come at a cost to the child, as it involved a desirable toy. Fifty-five percent of the children with ASD who did not provide help retrieved the toy but played with it themselves rather than giving it to the experimenter. In contrast, the target objects used by Liebal et al. (2008) were likely less interesting to the child (e.g., clothespin, pen) and potentially easier to part with (see also Paulus and Rosal-Grifoll, 2017). Indeed, the correlation between helping and sharing that was uniquely observed in the ASD group lends further support to the idea that the current task imposed unintended challenges associated with inhibiting a prepotent response, namely relinquishing a desired resource. Relatedly, because the experimenter was playing with the toy as opposed to using it in a more unambiguously goal directed manner – such as Liebal et al.’s (2008) experimenter who was using the pins to hang clothes – the experimenter’s need may have been less clear to the participants with ASD. This methodological limitation provides support for proposals regarding the multifaceted nature of prosocial motivation (e.g., Paulus, 2018).

### Sharing

Replicating past research, TD participants recognized another’s material desire and readily shared their treats when presented with unequal distributions (Dunfield et al., 2011; Dunfield and Kuhlmeier, 2013). When presented with the identical paradigm, 58% of 24-month-old offered to share their resources with a needy experimenter. Consistent with this observation, 78.6% of our TD participants shared in the experimental condition. To our knowledge, the present study is the first to experimentally evaluate (i.e., by including a control trial) preschoolers with ASD for their propensity to recognize a social partner’s lack of material need and then act to alleviate that need by sharing some of their own material resources.

In the present study, approximately one third of children with ASD shared with the experimenter when they had an abundance of treats but she had none. In contrast, no participants shared their snack when both parties had equal portions, suggesting at least some recognition of the experimenter’s material need in the former. Yet, relative to TD children, children with ASD shared at a much lower rate. This pattern of results is in striking contrast to Paulus and Rosal-Grifoll’s (2017) finding that participants with ASD tended to give most of their resources away. Interpreting this difference is difficult because it is impossible to tell whether the low sharing rates were due to difficulties in perspective taking or low motivation. To the extent that ASD participants in our sample recognized material desire, it remains unclear as to *how* the children determined that a need was present, given that they had multiple cues (i.e., an unequal distribution, an outstretched hand, and a negative facial expression) available. Indeed, explicit instructions to distribute the resources may help explain the high levels of generous behavior observed in previous research. Unfortunately, the nature of our design does not permit us to comment on the mechanism underlying the reduced rate of sharing in children with ASD, which may relate to obstacles in need-detection, a motivational component, or the capacity to produce sharing behavior.

Previous work with TD populations shows that even when children know that they should share, and expect others to share, they have difficulty enacting norms of fairness (Blake, 2018). Relatedly, behavioral control is importantly and uniquely associated with sharing over and above concerns about fairness in TD participants (Steinbeis and Over, 2017). As highlighted above, our results leave open the possibility that relatively lower rates of prosocial behavior in general in our ASD sample could be due to lower social motivation, or increased difficulty inhibiting prepotent responses. Future research can seek to better understand the intersecting influence of social cognition and motivation in the production of prosocial behavior.

### Comforting

Similar to past research, the TD participants readily recognized and appropriately responded to the experimenter’s emotional distress (Dunfield et al., 2011; Dunfield and Kuhlmeier, 2013). Specifically, previous research, using a shortened response period (i.e., 10 s) found no comforting in 24-month-old TD children (Dunfield et al., 2011), but with age, and a longer response period, 3- to 4-year-old TD children readily recognized and responded to another’s emotional distress (Dunfield and Kuhlmeier, 2013). Importantly, children with ASD did so as well. Indeed, a majority of children with ASD comforted the experimenter (65%), either verbally (42.90%) or physically (14.30%), and were as likely to do so as their TD peers. Despite the scarcity of comparative research on comforting in young children with ASD, and disparate findings within the field of empathy, the current results were unexpected given the literature that characterizes ASD children and adults as tending to be poor at identifying others’ emotional states (e.g., Dyck et al., 2001).

A counterintuitive, yet plausible, explanation is that having a reduced empathic response (Corona et al., 1998) may have actually benefitted children with ASD in the current study. While we take caution in interpreting the affective responses of our sample given that our measurement was only of observable/audible acts of comfort, children with ASD did not appear to treat the comforting trial as an emotionally laden task. The comforting behavior exhibited by children with ASD was largely instrumentally oriented (e.g., kissing the experimenter on the knee, asking if the experimenter needed a Band-Aid, providing direction) and lacked signs of personal distress. Thus, consistent with Corona et al. (1998), children with ASD in the current study did not appear averse to the situation itself, affording the opportunity to act in an other-oriented manner. Moreover, the automaticity of their responses suggests that these children with ASD had good knowledge of, or scripts for, what to do when someone is hurt, which were activated once the need for comfort was detected. Importantly, this tendency to engage in approach-oriented comforting behavior differed from the TD children who were more reserved in their provision of comfort. Specifically, none of the TD participants directly approached the experimenter; instead they tended to engage their caregiver or offer verbal reassurance.

Taken together, the experience of witnessing the experimenter hurt herself and determining an appropriate course of action may have been less emotionally taxing to children with ASD than TD children (for more discussion of the emotional cost of comforting, see Hoffman, 1982, 2000). It is possible that TD children may have experienced more emotional contagion when faced with the experimenter’s distress resulting in more personal distress and less other-oriented behavior (Eisenberg et al., 1991). This interpretation, if accurate, again draws upon the distinction between ‘empathy’ and ‘comforting’ in that the latter need not require one to share in the emotional experience of distress.

Comparing children with ASD’s responses to the three varieties of negative states – instrumental need, material desire, and emotional distress – may suggest an important insight into the combined effects of social cognitive understanding and motivation on the production of prosocial behavior in early childhood. Children with ASD may have perceived the comforting task as relatively ‘cost-free,’ as the experimenter could be comforted without having to relinquish ownership of a desired good. Specifically, to enact effective helping or sharing in the current tasks the child was required to relinquish control of desired goods (i.e., an action figure and preferred treats respectively); in contrast, there were no material costs associated with offering the pained experimenter verbal or physical support. Documented deficits in inhibitory control in ASD populations (Hill, 2004) support the proposal that the “cost” of helping and sharing in the current task may be higher for participants with ASD than TD participants. Because both the behavioral and emotional costs of responding to emotional distress appear to be lower for ASD participants than TD participants, especially relative to instrumental needs and material desire, it is possible that comforting behaviors most clearly reflect the other-oriented tendencies of children with ASD.

Another possibility is that the parameters of the comforting task facilitated the detection of the experimenter’s need. Multiple factors can add to the complexity of this task, including the complexity of the need (instrumental, material, emotional) and the number of cues that are at children’s disposal (verbal, non-verbal, situational; e.g., Svetlova et al., 2010). The comforting paradigm used in the present study offered children a clear verbal cue as to what had happened to the experimenter (“Oh! My knee. I banged my knee!”). Though the required intervention was not verbalized, this verbal marker of distress may have increased the saliency of the fact that the experimenter was hurt. Best efforts were made to ensure each of the three tasks were comparable and verbalizations were made during the helping and sharing trials, but they did not directly say what was ‘wrong,’ leaving children to rely on the non-verbal cues offered by the experimenter instead. Given that children with ASD are challenged in their understanding of non-verbal cues (Dyck et al., 2001), they may have been disadvantaged at detecting the experimenter’s need in the helping and sharing trials relative to the comforting trials. The success of children with ASD on the comforting task may then be associated with the increased saliency of the experimenter’s need, which, in turn, aided in their extraction of pertinent information about the situation and potentially activated a repertoire of scripted comforting responses. Under this account, knowing how to comfort the experimenter may not have been achieved through a true comprehension of the complex emotional need associated with being hurt, but through previous scaffolding of what one should do when a person indicates that they are hurt. Alternatively, participants had a longer response window in the comforting task relative to the helping and sharing tasks, and the relative performance of the two groups across the three tasks may suggest that prosocial behaviors take longer to mount for children with ASD. Future research is necessary to further investigate these complimentary interpretations.

### Future Directions and Limitations

While the present study advances our understanding the prosocial tendencies of young children with ASD, and the role of social cognition and motivation in the production of prosocial behavior, it represents only the beginning stages of a movement toward properly appreciating what forms of prosocial behavior children with ASD can and will engage in, what cues are necessary to detect a social partner’s need, and what developmental pre-requisites are needed to enable engagement in effective other-oriented behavior. Most likely, the constellation of results obtained in the present study suggests that a myriad of factors are implicated in the propensity of children with and without ASD to act prosocially.

The present study is exploratory and contains some broad limitations that highlight areas that may be addressed in future research. First, knowing how to act on another’s behalf does not necessarily equate to *understanding* that person’s need. Instead, having a repertoire of learned behaviors, or scripts, to draw upon when prosocial behavior is warranted may be sufficient. Likewise, the absence of prosocial behavior in some of our participants must not be interpreted as a lack of understanding about what to do, as multiple factors that were not measured here may have inhibited children’s ability to *demonstrate* and *communicate* their understanding (e.g., motivation, use of gestures, motor planning, etc.). Finally, as recently discussed, it is notoriously difficult to determine the motivation that underlies atypical social cognitive performance in young children with ASD (Jaswal and Akhtar, 2018). Indeed, the propensity for children with ASD to readily offer an experimenter support when the costs of doing so are low suggest that even young children with ASD are not disinterested in the welfare of others. Future research employing novel physiological designs (e.g., pupil dilation, Hepach et al., 2017) may shed additional light on this important, open question.

## Conclusion

The current study has provided an initial demonstration that young children with ASD are able to distinguish situations where need is warranted from when it is not and appear to be able to tap into pertinent knowledge about a person’s intentions and desires. However, when the cost of engaging in prosocial behavior is high, children with ASD may be less inclined to engage in the behavior. Both the capacity to recognize another’s need *and* the drive to act on behalf of another appear to play important roles in the production of prosocial behavior. Other variables, including the saliency of the indicators, behavioral control, and learned behaviors may also support the prosocial performance in children with ASD and require further exploration. Future investigation is needed to systematically delineate the individual and environmental correlates of prosocial behavior in this population and how this population can inform our understanding of the pervasive tendency for *Homo sapiens* to act on behalf of others.

## Ethics Statement

This study was carried out in accordance with the recommendations of the Queen’s University General Research Ethics board. Parents of the participants provided written informed consent, all subjects additionally provided verbal or non-verbal assent. The protocol was approved by the Queen’s University General Research Ethics board.

## Author Contributions

KD, LB, EK, and VK designed the study. LB collected the data and conducted preliminary analyses. KD conducted the primary analyses. KD, LB, EK, and VK all contributed to the writing of the current manuscript.

## Conflict of Interest Statement

The authors declare that the research was conducted in the absence of any commercial or financial relationships that could be construed as a potential conflict of interest.
